# Hippocampal Synaptic Dysfunction in a Mouse Model of Huntington Disease Is Not Alleviated by Ceftriaxone Treatment

**DOI:** 10.1523/ENEURO.0440-19.2020

**Published:** 2020-05-21

**Authors:** Crystal M. Wilkie, Jocelyn R. Barnes, Cherry-Lynn M. Benson, Kyle J. Brymer, Firoozeh Nafar, Matthew P. Parsons

**Affiliations:** Division of Biomedical Sciences, Faculty of Medicine, Memorial University, St. John’s, Newfoundland A1B 3V6, Canada

**Keywords:** GLT-1, glutamate, glutamate uptake, Huntington's disease, iGluSnFR, plasticity

## Abstract

Glutamate transporters, particularly glutamate transporter 1 (GLT-1), help to prevent the adverse effects associated with glutamate toxicity by rapidly clearing glutamate from the extracellular space. Since GLT-1 expression and/or function are reduced in many neurodegenerative diseases, upregulation of GLT-1 is a favorable approach to treat the symptoms of these diseases. Ceftriaxone, a β-lactam antibiotic reported to increase GLT-1 expression, can exert neuroprotective effects in a variety of neurodegenerative diseases; however, many of these diseases do not exhibit uniform brain pathology. In contrast, as a drug that readily crosses the blood–brain barrier, ceftriaxone administration is likely to increase GLT-1 levels globally throughout the neuroaxis. In Huntington disease (HD), low GLT-1 expression is observed in the striatum in postmortem tissue and animal models. While ceftriaxone was reported to increase striatal GLT-1 and ameliorate the motor symptoms in a mouse model of HD, the extrastriatal effects of ceftriaxone in HD are unknown. Using electrophysiology and high-speed imaging of the glutamate biosensor iGluSnFR, we quantified real-time glutamate dynamics and synaptic plasticity in the hippocampus of the Q175FDN mouse model of HD, following intraperitoneal injections of either saline or ceftriaxone. We observed an activity-dependent increase in extracellular glutamate accumulation within the HD hippocampus, which was not the result of reduced GLT-1 expression. Surprisingly, ceftriaxone had little effect on glutamate clearance rates and negatively impacted synaptic plasticity. These data provide evidence for glutamate dysregulation in the HD hippocampus but also caution the use of ceftriaxone as a treatment for HD.

## Significance Statement

Huntington disease (HD) is an inherited neurodegenerative disease. In addition to the debilitating motor symptoms, HD is commonly associated with burdensome cognitive impairments. Here, we used a mouse model of HD to show that in a region essential for cognition, the hippocampus, excessive levels of the neurotransmitter glutamate accumulate during neural activity. While required for rapid cellular communication, too much glutamate impairs synapse strengthening and negatively impacts cellular health. Glutamate accumulation in the HD hippocampus appeared not to be due to altered expression of glutamate transporter-1, a highly expressed protein in the brain that controls glutamate levels. This is the first study to show abnormal glutamate accumulation in the HD hippocampus, which may underlie the devastating cognitive symptoms associated with HD.

## Introduction

Glutamate is the most abundant neurotransmitter in the brain and is essential for rapid cell-to-cell communication. Paradoxically, glutamate can also exert toxic effects on neurons, and increasing evidence suggests that the toxic actions of glutamate occur at least in part through the activation of extrasynaptically located NMDA receptors (NMDARs; Hardingham and Bading, 2010; Parsons and Raymond, 2014). Fortunately, the brain is equipped with a robust and efficient glutamate transporter system that maintains low levels of ambient extracellular glutamate and helps to prevent excessive glutamate spillover to extrasynaptic sites during neurotransmission (Danbolt, 2001). Glutamate transporter 1 (GLT-1) is an essential excitatory amino acid transporter protein that is expressed in high concentrations throughout much of the neuroaxis (Lehre and Danbolt, 1998). While GLT-1 has been detected in neurons, it is primarily found on astrocytic membranes (Rothstein et al., 1994; Furness et al., 2008). Knocking out GLT-1 selectively in astrocytes recapitulates the lethal seizure phenotype observed following global GLT-1 knockout (Tanaka et al., 1997; Petr et al., 2015). GLT-1 plays an important role in regulating extracellular glutamate dynamics following synaptic release in the healthy brain (Pinky et al., 2018), and GLT-1 expression and/or function is reduced in a variety of CNS conditions (Rothstein et al., 1995; Masliah et al., 1996; Liévens et al., 2001; Jacob et al., 2007). Therefore, pharmacological upregulation of GLT-1 has been suggested as a promising therapeutic strategy for a wide variety of neurologic conditions including several neurodegenerative diseases, addiction, ischemia, chronic pain, and traumatic brain injury (Yimer et al., 2019).

Ceftriaxone (Cef) is a Food and Drug Administration-approved β-lactam antibiotic known to increase GLT-1 expression through the nuclear factor-κB pathway (Rothstein et al., 2005; Lee et al., 2008). Evidence supporting a neuroprotective effect of ceftriaxone has been observed in mouse models of a diverse set of neurodegenerative diseases including Parkinson’s disease, Alzheimer’s disease, amyotrophic lateral sclerosis and Huntington disease (HD), to name a few (Yimer et al., 2019). In theory, pharmacologically increasing GLT-1 expression in neurologic conditions associated with glutamate toxicity resulting from low GLT-1 levels is indeed an attractive therapeutic strategy. However, as a cephalosporin that crosses the blood–brain barrier (Spector, 1987), systemic administration of ceftriaxone is likely to exert an effect throughout much of the neuroaxis. In contrast, most CNS conditions are associated with varying degrees of pathology among different brain regions, and increasing GLT-1 expression in regions where the levels and function of the transporter are unperturbed may be associated with adverse side effects. Indeed, ceftriaxone administration to wild-type (WT) rats was reported to negatively impact hippocampal-dependent learning and memory (Matos-Ocasio et al., 2014) and mossy fiber-CA3 long-term depression (Omrani et al., 2009).

HD is a devastating neurodegenerative disease caused exclusively by a CAG repeat expansion in the gene encoding the huntingtin protein. Although HD is now recognized as a brain-wide disease (Ross et al., 2014), the HD-causing mutation preferentially affects striatal medium spiny neurons, which results in the devastating motor symptoms of the disease. It has been shown that GLT-1 expression is reduced in the striatum of HD mouse models and in postmortem HD striatal tissue (Liévens et al., 2001; Behrens et al., 2002; Miller et al., 2008; Faideau et al., 2010). Interestingly, ceftriaxone increased striatal GLT-1 levels and ameliorated the motor deficits in the aggressive R6/2 mouse model of HD. However, HD is also associated with extremely burdensome cognitive decline (Paulsen, 2011), and clear deficits in hippocampal synaptic plasticity and hippocampal-dependent learning and memory have been observed in numerous mouse models of HD (Usdin et al., 1999; Murphy et al., 2000; Lynch et al., 2007; Brito et al., 2014; Kolodziejczyk et al., 2014; Giralt et al., 2017; Quirion and Parsons, 2019). While the literature on GLT-1 function in the HD hippocampus is sparse, one report did show that GLT-1 expression is normal in the hippocampus of the YAC128 mouse model of HD up to 12 months of age (Huang et al., 2010). While ceftriaxone may have beneficial effects on striatal pathology and motor symptoms in HD, its putative effect on hippocampal function remains to be seen. Here, we used high-speed imaging of a glutamate biosensor and electrophysiology to quantify hippocampal glutamate dynamics and synaptic plasticity, and their response to ceftriaxone treatment, in the Q175FDN knock-in mouse model of HD. Our results demonstrate that while excessive extracellular glutamate accumulation can be detected in the HD hippocampus following certain presynaptic activity patterns, it is not a result of low GLT-1 expression. Furthermore, ceftriaxone had surprisingly little effect on functional measures of glutamate dynamics and negatively impacted hippocampal synaptic plasticity.

## Materials and Methods

*Animals.* In the present study, we used heterozygous (Het) Q175FDN mice (Southwell et al., 2016) and their WT littermates, bred within the animal care facility of Memorial University. DNA sequencing (Laragen) was performed on a subset of samples and mice with repeat lengths ∼205 were selected as breeders. All mice were group housed in ventilated cage racks and kept on a 12 h light/dark cycle (lights on at 7:00 A.M.) with food and water available *ad libitum*. Both male and female mice were used in equal numbers in the present study. No sex differences were observed in our experimental measures, and data from male and female mice were combined. All procedures followed the guidelines set by the Canadian Council on Animal Care and were approved by the Institutional Animal Care Committee of Memorial University.

*Stereotaxic surgery.* At 5–6 months of age, mice were anesthetized with isoflurane (3% induction, 1.5–2% maintenance) and injected with 2 mg/kg, s.c., meloxicam and 0.1 ml/0.2% lidocaine underneath the scalp before the surgical procedure. A hand drill was used to drill a small hole at the desired coordinates, and a Neuros 7002 Hamilton Syringe was used with an infusion pump (Pump 11 Elite Nanomite, Harvard Apparatus) to inject 1 μl of AAV1.hSyn.iGluSnFr.WPRE.SV40 into the hippocampus (injection rate, 2 nl/s). We used the following coordinates with respect to distance from bregma: 2.6 mm posterior, 2.4 mm lateral, 1.2–1.4 mm ventral to brain surface. pAAV.hSyn.iGluSnFr.WPRE.SV40 was a gift from Loren Looger (viral prep #98 929-AAV1, Addgene; http://n2t.net/addgene:98929; RRID:Addgene_98929). The syringe was left in place for at least 5 min following the injection. The incision was then sutured, and 0.5 ml of 0.9% saline was administered subcutaneously. Mice were warmed on a heating pad for ∼30 min and then returned to the ventilated cage racks.

*Slice preparation.* Approximately 2–3 weeks following iGluSnFR injection, mice were injected daily for 7 d with ceftriaxone (200 mg/kg, i.p.). Twenty-four hours after the last injection, when mice were 6–7 months of age, mice were anesthetized with isoflurane and decapitated, and the brain was quickly removed and placed in ice-cold oxygenated (95% O_2_/5% CO_2_) slicing solution consisting of the following (in mm): 125 NaCl, 2.5 KCl, 25 NaHCO_3,_ 1.25 NaH_2_PO_4_, 2.5 MgCl_2_, 0.5 CaCl_2_, and 10 glucose. Transverse slices (350 μm) containing the hippocampus were cut using a Leica VT1000 S Vibratome. Slices were recovered in artificial CSF (ACSF) at room temperature for at least 60–90 min before imaging and electrophysiology experiments. ACSF consisted of the following (in mm): 125 NaCl, 2.5 KCl, 25 NaHCO_3_, 1.25 NaH_2_PO_4_, 1.0 MgCl_2_, 2.0 CaCl_2_, and 10 glucose.

*iGluSnFR imaging and analysis.* Slices from 6- to 7-month-old mice expressing iGluSnFR were transferred to a recording chamber, and a peristaltic pump (MP-II, Harvard Apparatus) was used to perfuse oxygenated ACSF at a flow rate of 1.5–2 ml/min. ACSF was maintained at 25°C using an in-line heater and temperature controller (TC-344C, Harvard Apparatus). A glass stimulating electrode was placed in the Schaffer collateral pathway, ∼50–100 μm below the slice surface. Clampex software and a Digidata 1550A (Molecular Devices) were used to control LED illumination (Lumen 300, Prior Scientific), image acquisition through an EM-CCD camera (Andor iXon Ultra 897, Oxford Instruments), and electrical stimulation with an Iso-flex Stimulus Isolator (A.M.P.I.). iGluSnFR responses to synaptic stimulation were imaged using an Olympus BX61 upright microscope and a 4×/0.28 numerical aperture objective (Olympus). Images were captured at 205 frames per second using Andor Solis software (Oxford Instruments). Image binning of 4 × 4 was used. iGluSnFR responses were evoked in each slice with either a single train of high-frequency stimulation (HFS; 100 pulses over 1 s) or theta burst stimulation (TBS; 10 bursts of four pulses at 100 Hz, separated by a 200 ms interburst interval). Stimulus intensity was set at 50 μA for these experiments, which represents a stimulus intensity that typically evokes a response that is ∼30–40% of the maximal response on this system. After receiving either HFS or TBS, the slice was discarded.

iGluSnFR responses to synaptic stimulation were quantified by first applying bleach correction using the bleach correction plugin in FIJI software. Bleaching was kept to a minimum by limiting the exposure of the slice to blue light, and any bleaching observed was readily corrected in FIJI. Following bleach correction, a 10 × 10 pixel region of interest (1 pixel at 4 × 4 binning = 15.6 μm) was placed 150–200 μm away from the stimulating electrode, toward CA1, in the stratum radiatum. For each image frame, the average iGluSnFR intensity was calculated within this region of interest. Arbitrary fluorescence intensity units were converted to %Δ*F*/*F* (percentage change in fluorescence intensity relative to the basal fluorescence intensity) using the VSD signal processor plugin. iGluSnFR sustain was calculated by dividing the %Δ*F*/*F* at the end of HFS by the response peak. Decay tau and the area under the curve were calculated using GraphPad Prism 8. For decay tau calculations, response decays were fit with a single-exponential nonlinear curve in GraphPad Prism 8. For image presentation in the figures, noise was reduced using a Gaussian blur filter (2 pixels), the scale was set from 1–6% Δ*F*/*F*, and the “fire” heatmap was applied. For the *x-y* maximum projection images shown, 4 × 4 scaling was applied with bilinear interpolation. The “volume viewer” plugin was used to visualize the response along the *z*-axis (time).

*Electrophysiology.* For electrophysiological recordings of basal excitability and long-term potentiation (LTP), acute hippocampal slices, obtained from 6- to 7-month-old mice, were positioned on a probe consisting of an 8 × 8 array of 64 electrodes, each spaced 150 μm apart (MED-P515A probe, Alpha MED Scientific). Before use, each MED64 probe was treated overnight with 0.1% polyethyleneimine in 25 mm borate buffer, adjusted to a pH of 8.4 with HCl. Once a slice was placed in the probe, the hippocampus was positioned over the electrode array with the visual aid of a USB digital camera imaging from the bottom side of the probe (USB2-MICRO-250X, Plugable). Once positioned with electrodes covering CA3 and CA1 subregions of the hippocampus, the probe was placed into the MED64 ThermoConnector (MED-CP04) and perfused with oxygenated ACSF using a peristaltic pump. The flow rate was set to 1.5–2 ml/min, and the temperature of the ACSF was maintained at 25°C using a temperature controller. The slice was left in the system to acclimatize for at least 15 min before stimulation.

After 15 min, an electrode that was positioned within the Shaffer collateral projection from CA3 to CA1 was selected to be the stimulating electrode. Mobius software was used to select the stimulating electrode and to monitor responses in the remaining 63 electrodes. An input/output curve was performed by evoking field EPSPs (fEPSPs) starting at a stimulus intensity of 10 μA and increasing by 5 μA steps. From these input–output curves, we determined the stimulation intensity that elicited 30–40% of the maximal fEPSP response. This stimulation intensity was then used as the stimulus intensity for the subsequent LTP experiment. For LTP experiments, slices were stimulated with single pulses (0.2 ms width) every 20 s. After a stable baseline was achieved, HFS was applied to induce LTP. HFS consisted of 100 pulses at 100 Hz. Following HFS, single fEPSPs were evoked every 20 s for an additional 60 min. For fEPSP analysis, we selected three adjacent electrodes within stratum radiatum that exhibited the most robust response during baseline recordings, as determined by the slope of the first 1–2 ms of the fEPSP slope. The fEPSP slope at these three locations within stratum radiatum was then averaged together to generate a single fEPSP slope in stratum radiatum in response to each single stimulus applied to the Schaffer collaterals. The percentage of potentiation was determined by averaging the fEPSP response size from 55 to 60 min following HFS, and by expressing it as a percentage increase from the average response of the baseline. Measurements of paired-pulse ratios were conducted on a separate conventional electrophysiological recording system exactly as described previously (Barnes et al., 2020).

*Western blotting.* For Western blot quantification of GLT-1 protein expression, the hippocampus of the noninjected hemisphere in iGluSnFR-injected animals (6–7 months of age) was dissected and homogenized in 200–300 μl of lysis buffer containing protease and phosphatase inhibitors (Roche). The supernatant was collected, and the protein concentration was determined using BCA standards. Fifty micrograms of protein was added to each lane of a 10% SDS-PAGE gel for electrophoresis before being transferred to a nitrocellulose membrane. Primary antibodies for GLT-1 (1:1000; EI, mouse-monoclonal, Santa Cruz Biotechnology) and actin (1:1000; C4, mouse-monocolonal, Santa Cruz Biotechnology), and a goat anti-mouse IgG-HRP secondary antibody (monoclonal; 1:5000; catalog #sc-2005, Santa Cruz Biotechnology) were used. Blots were developed using a chemiluminescent HRP substrate (catalog #WBKLS0100, lot no. 1712501, Millipore). Band densities were quantified in ImageJ, and GLT-1 band densities were normalized to actin.

*Statistics.* Statistical tests used included two-way ANOVA, repeated-measures two-way ANOVA, unpaired *t* test (two-tailed), and paired *t* test (two-tailed). *p* values <0.05 were considered significant. For all two-way ANOVA analyses, the *p* values for the main effects (genotype and treatment) and for the interaction effects are clearly noted in the results text and in the corresponding figure panel. As the main research question focused on the effect of ceftriaxone on glutamate dynamics, Sidak *post hoc* tests were performed only when a main treatment or interaction effect was found. For imaging and LTP experiments, the reported *n* values represent the number of slices obtained from at least five animals in each condition. For all Western blot data, reported *n* values refer to the number of animals used in each condition.

## Results

### Glutamate dynamics are altered in the Q175FDN hippocampus in response to high-frequency stimulation

**Figure 1.**
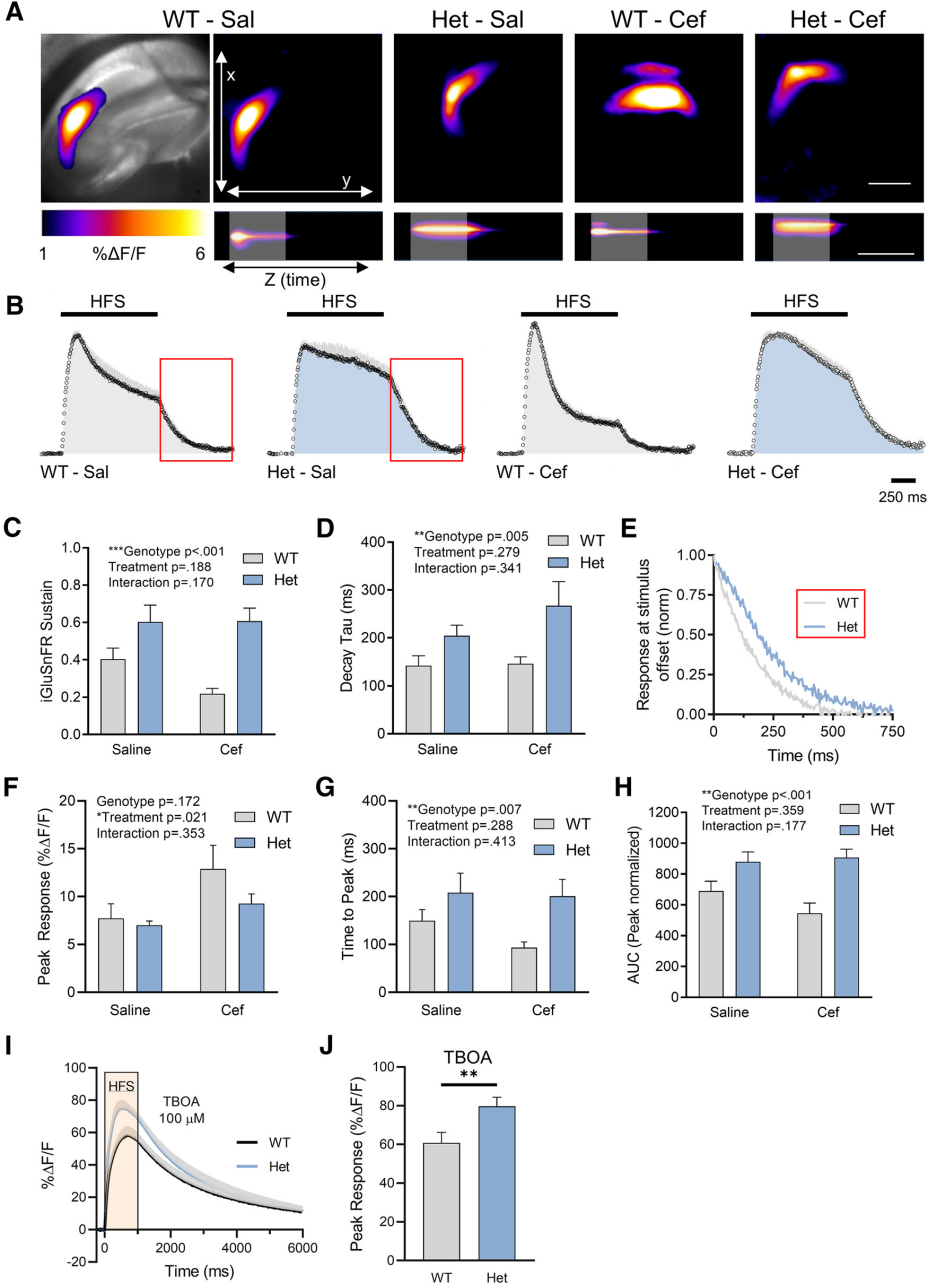
HFS results in excess glutamate accumulation in the Q175FDN Het hippocampus that is not alleviated by ceftriaxone. ***A***, Representative maximum projection images of the iGluSnFR response to HFS in WT-saline (Sal), Het-Sal, WT-Cef, and Het-Cef conditions. A bright-field image depicting the field of view containing the hippocampus is shown on the top left for the WT-Sal example. Scale bar, top right (top five images), 500 μm. The bottom panels represent the response to HFS over time (*z*-axis). Gray shading indicates the timing of HFS. Scale bar, bottom right, 1 s. ***B***, Mean iGluSnFR responses (±SEM) in each group during HFS. The red box represents the portion of the response at stimulus offset used for the decay tau analysis in ***D***. Gray shading above the response indicates SEM. The 1 s of HFS is indicated by the black horizontal bar above the response, and the shading underneath each response is representative of the area under the curve. Responses are normalized to the peak. ***C***, Analysis of iGluSnFR sustain in WT and Het mice treated with Sal or Cef. ***D***, Decay tau comparisons for each condition. ***E***, Representative response at the end of stimulation showing the difference in iGluSnFR decay between WT-Sal and Het-Sal. ***F–H***, Peak (***F***), time to reach the peak (***G***), and the area under the curve (AUC; ***H***) in WT and Het mice treated with Sal or Cef. ***I***, ***J***, iGluSnFR responses (***I***) and peak values (***J***) in WT and Het slices exposed to 100 μm TBOA. All data are presented as the mean ± SEM. *p < 0.05, **p < 0.01, ***p < 0.001.

We treated iGluSnFR-injected WT and Q175FDN heterozygous HD mice with either saline or ceftriaxone. Acute brain slices containing the hippocampus were obtained, and iGluSnFR responses in CA1 stratum radiatum were quantified following electrical stimulation (HFS; 100 pulses in 1 s) of the Schaffer collaterals. In addition to being a commonly used LTP induction paradigm, HFS is expected to challenge the glutamate uptake system in the hippocampus by overwhelming glutamate transporters (Pinky et al., 2018). In WT mice treated with saline, HFS produced an iGluSnFR response that peaked at 149.6 ± 23.3 ms (*n* = 11) into the stimulation paradigm (Fig. 1*A*). After responses peaked, and in the continued presence of a single HFS train, the iGluSnFR response then decreased to a value less than half of the peak by the end of HFS. When HFS was terminated, the iGluSnFR signal then rapidly fell back to baseline levels (Fig. 1*A*,*B*). Mean ± SEM iGluSnFR responses to HFS are shown in Figure 1*B* for all conditions (WT-saline, *n* = 11; Q175FDN-saline, *n* = 10; WT-ceftriaxone, *n* = 9; Q175FDN-ceftriaxone, *n* = 10). The observed reduction in response size throughout the latter portions of HFS is consistent with a recent study that used the ultrafast iGlu*_u_* glutamate sensor to demonstrate that short-term depression of glutamate release occurs when Schaffer collaterals are stimulated at 100 Hz (Helassa et al., 2018). Therefore, we quantified “iGluSnFR sustain” as a putative measure of the magnitude of presynaptic depression during HFS, where higher sustain is suggestive of less presynaptic depression.

Interestingly, the presynaptic depression observed in slices from WT mice was not as pronounced in slices from Q175FDN mice, as indicated by higher mean iGluSnFR sustain values recorded from Q175FDN mice and a statistically significant genotype effect (Fig. 1*C*; two-way ANOVA; genotype, *p* < 0.001). This result suggests that normally occurring presynaptic depression is impaired in the Q175FDN hippocampus, thereby promoting excess extracellular glutamate accumulation during HFS. Ceftriaxone was without effect on the high sustain values observed in Q175FDN mice, as no significant treatment or interaction effect was observed (Fig. 1*C*; two-way ANOVA; treatment, *p* = 0.188; interaction, *p* = 0.170). However, it is worth noting that when WT mice were analyzed independently of the Q175FDN data, iGluSnFR sustains were significantly reduced following ceftriaxone treatment (unpaired *t* test, *p* = 0.017). Thus, ceftriaxone can reduce glutamate accumulation during HFS in WT mice, but provides no beneficial effect in the context of HD.

Next, to determine whether relative differences could be observed in glutamate clearance rates after electrical afferent stimulation was terminated, we quantified the decay tau of iGluSnFR as it returned to baseline after HFS had ended. We found that after HFS, glutamate clearance was slower overall in Q175FDN mice compared with WT mice, and a significant genotype effect was found (Fig. 1*D*,*E*; two-way ANOVA; genotype, *p* = 0.005). Although ceftriaxone is widely used to increase glutamate transporter expression, glutamate clearance rates were not significantly affected by ceftriaxone treatment, regardless of genotype (Fig. 1*D*; two-way ANOVA; treatment, *p* = 0.279; interaction, *p* = 0.341). We observed no genotype effect on the maximum amount of glutamate released during HFS, as measured by the peak iGluSnFR response (Fig. 1*F*; two-way ANOVA; genotype, *p* = 0.172). Unexpectedly, ceftriaxone increased the size of iGluSnFR responses in WT mice (Fig. 1*F*; two-way ANOVA, treatment, *p* = 0.021; Sidak’s multiple-comparisons test, *p* = 0.041 for WT-saline vs WT-Cef; *p* = 0.536 for Q175FDN-saline vs Q175FDN-Cef). It took significantly longer to reach a peak in Q175FDN mice (Fig. 1*G*; two-way ANOVA; genotype, *p* = 0.007), but ceftriaxone had no significant effect on the time to peak (Fig. 1*G*; two-way ANOVA; treatment, *p* = 0.288; interaction, *p* = 0.413). We also observed a significant genotype difference in the area under the curve of the iGluSnFR profile, which was larger in Q175FDN mice but again was unaffected by ceftriaxone treatment (Fig. 1*H*; genotype, *p* < 0.001; treatment, *p* = 0.359; interaction, *p* = 0.177). Together, these data suggest that impaired presynaptic depression and slow glutamate clearance contribute to excessive extracellular glutamate accumulation in the Q175FDN hippocampus in response to HFS. However, these observed effects were not alleviated by ceftriaxone treatment.

The higher iGluSnFR sustain observed in Q175FDN mice (Fig. 1*C*) could be accounted for by more presynaptic glutamate release throughout the HFS protocol (i.e., less presynaptic depression). However, the iGluSnFR profile during HFS is likely to reflect a combination of the amount of ongoing glutamate release as well as the rate of glutamate uptake during this period of stimulation. To remove contributions from the latter, we performed a set of experiments in slices from WT and Q175FDN mice and evoked iGluSnFR signals with HFS as above, but this time in the presence of a saturating concentration (100 μm) of the glutamate transporter inhibitor DL-TBOA (DL-threo-beta-benzyloxyaspartate). d-APV (50 μm) and DNQX (20 μm) were added to the ACSF to prevent excitotoxicity. In the absence of transporter-mediated glutamate uptake, HFS resulted in significantly larger iGluSnFR responses in Q175FDN slices compared with WT slices (Fig. 1*I*,*J*; unpaired *t* test, *p* = 0.016). This finding suggests that elevated glutamate levels can be reached in HD tissue independent of glutamate uptake, thereby supporting the interpretation that presynaptic depression during HFS is reduced in the Q175FDN hippocampus.

### Glutamate dynamics are normal in the HD hippocampus in response to theta-burst stimulation

**Figure 2.**
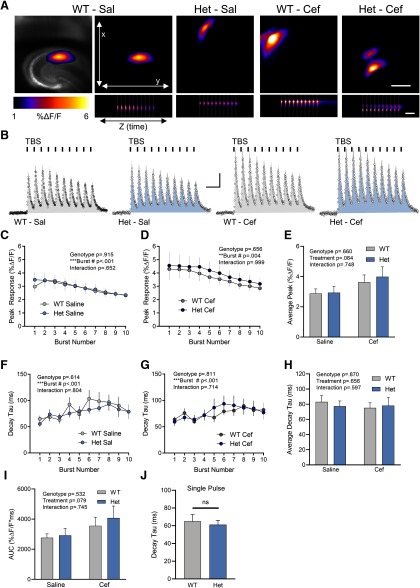
Glutamate dynamics in response to TBS are normal in the Q175FDN Het hippocampus. ***A***, Representative maximum projection images of the iGluSnFR response to TBS in WT-saline (Sal), Het-Sal, WT-Cef, and Het-Cef conditions. A bright-field image depicting the field of view containing the hippocampus is shown on the top left for the WT-Sal example. Scale bar, top right (top five images), 500 μm. The bottom panels represent the response to TBS over time (*z*-axis). Gray shading indicates the timing of the 10 bursts of TBS. Calibration, bottom right: 1 s. ***B***, Mean iGluSnFR responses (±SEM) in each group during TBS. Gray shading above the response indicates SEM. Each burst associated with TBS is indicated by a vertical black bar above the response, and the shading underneath each response is representative of the area under the curve. Calibration (all four responses): 1% Δ*F*/*F*; 400 ms. ***C***, ***D***, Mean (±SEM) peak response to each of the 10 bursts associated with TBS in WT and Het mice treated with Sal (***C***) or Cef (***D***). ***E***, Comparison of average peak over TBS in WT and Het treated with Sal or Cef. ***F***, ***G***, Decay tau for each of the 10 bursts associated with TBS in WT and Het mice treated with Sal (***F***) or Cef (***G***). ***H***, Comparison of the average decay tau throughout TBS in WT and Het treated with Sal or Cef. ***I***, Comparison of AUC in WT and HET mice treated with Sal or Cef. ***J***, iGluSnFR decay tau values evoked by a single pulse. All data are presented as the mean ± SEM; **p < 0.01, ***p < 0.001. ns, non-significant.

TBS, another commonly used LTP induction paradigm, consists of multiple bursts of activity separated by an interburst interval of 200 ms. Each burst consists of four pulses at 100 Hz, which in and of itself is insufficient to overwhelm the glutamate transporters in the hippocampus (Diamond and Jahr, 2000; Pinky et al., 2018), and the interburst interval of 200 ms is sufficient to restore any activity-dependent slowing of glutamate clearance (Armbruster et al., 2016). Therefore, it is expected that TBS places substantially less demand on the glutamate transporter system compared with the incessant train of 100 pulses associated with HFS. As altered glutamate dynamics were observed in the Q175FDN hippocampus following HFS, we next asked whether similar effects could be seen with the less demanding TBS protocol. In hippocampal slices from WT mice treated with saline, TBS resulted in a clear iGluSnFR response with 10 distinct peaks that were time locked to the 10 bursts associated with the TBS paradigm (Fig. 2*A*,*B*; WT-saline, *n* = 10; WT-ceftriaxone, *n* = 10; Q175FDN-saline, *n* = 10; Q175FDN-ceftriaxone, *n* = 9). Similar iGluSnFR responses were observed when comparing WT and Q175FDN mice treated with saline (Fig. 2*C*; repeated-measures two-way ANOVA: genotype, *p* = 0.915; burst number, *p* < 0.001; interaction, *p* = 0.652) as well as when comparing WT and Q175FDN mice treated with ceftriaxone (Fig. 2*D*; repeated-measures two-way ANOVA: genotype, *p* = 0.656; burst number, *p* = 0.004; interaction, *p* = 0.999). iGluSnFR response peaks were not different between the two genotypes, and while responses tended to be larger in ceftriaxone-treated mice, this did not reach statistical significance (Fig. 2*E*; two-way ANOVA; genotype, *p* = 0.660; treatment, *p* = 0.064; interaction, *p* = 0.748). Next, we analyzed iGluSnFR decay tau for each of the 10 iGluSnFR transients associated with each burst of TBS. No genotype differences were observed in iGluSnFR decay tau across the 10 distinct bursts in saline-treated mice (Fig. 2*F*; repeated-measures two-way ANOVA: genotype, *p* = 0.614; burst number, *p* < 0.001; interaction, *p* = 0.804). Similarly, no genotype differences were observed in iGluSnFR decay tau across the 10 distinct bursts in ceftriaxone-treated mice (Fig. 2*G*; repeated-measures two-way ANOVA: genotype, *p* = 0.811; burst number, *p* < 0.001; interaction, *p* = 0.714). When all 10 decay taus were averaged for each slice, we also found no significant genotype, treatment, or interaction effects using a two-way ANOVA (Fig. 2*H*; two-way ANOVA; genotype, *p* = 0.870; treatment, *p* = 0.656; interaction, *p* = 0.597). No significant genotype or treatment (i.e., saline vs ceftriaxone) effects were observed when we quantified the area under the curve of the iGluSnFR response throughout the entire TBS, although there was a trend toward larger areas in ceftriaxone-treated mice (Fig. 2*I*; genotype, *p* = 0.532; treatment, *p* = 0.079; interaction, *p* = 0.745). Together, these data suggest that the Q175FDN hippocampus exhibits activity-dependent alterations in extracellular glutamate accumulation, with normal iGluSnFR profiles during TBS but abnormal responses during HFS. In agreement, we found no evidence of impaired glutamate dynamics when glutamate release was evoked by a single pulse (Fig. 2*J*; WT, *n* = 12; Het, *n* = 14; unpaired *t* test, *p* = 0.674).

### Ceftriaxone negatively impacts LTP at CA3–CA1 synapses

**Figure 3.**
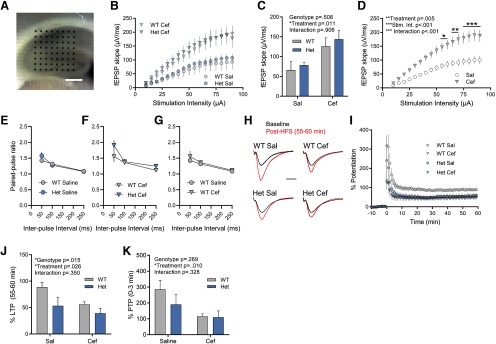
Ceftriaxone increases CA3–CA1 basal excitability and impairs synaptic plasticity. ***A***, Representative image of the multielectrode array used to stimulate and record hippocampal responses. Scale bar, 450 μm. ***B*–*D***, Input–output curve measuring fEPSP responses to increasing stimulus intensities in WT and Het mice treated with saline (Sal) or Cef. Mean fEPSP slopes at 50 μA are shown in ***C*** to compare the effect of treatment and genotype on the fEPSP. Data for Het and WT responses were grouped in ***D*** to demonstrate the effect of ceftriaxone treatment on fEPSP slope. Sidak’s multiple-comparisons test showed significant *post hoc* effects at 55–60 μA (*p < 0.05), 65–70 μA (**p < 0.01), and 75–90 μA (***p < 0.001). ***E–G***, Paired-pulse ratios at three different interpulse intervals for the indicated genotypes and treatments. ***H***, ***I***, LTP in WT and Het mice treated with Sal or Cef. Representative fEPSP traces before (black) and 55–60 min after LTP induction (red) are shown in ***H***. fEPSP traces are scaled so that the baseline peak is matched for each trace. Scale bar, 10 ms. High-frequency stimulation is administered at time = 0 in ***I***. ***J***, LTP expressed as the percentage potentiation 55–60 min following high-frequency stimulation. ***K***, Post-tetanic potentiation expressed as the percentage of potentiation 0–3 min following high-frequency stimulation. All data are presented as the mean ± SEM. *p < 0.05, **p < 0.01, ***p < 0.001.

We previously demonstrated that 6-month-old heterozygous Q175FDN mice, the age also used in the present study, exhibit an impairment in LTP induced by HFS, but not by TBS (Quirion and Parsons, 2019). Furthermore, the glutamate imaging results of the present study demonstrate that excessive glutamate accumulation occurs in the Q175FDN hippocampus during HFS but not TBS. While ceftriaxone was unable to restore HFS-evoked glutamate dynamics to WT levels, we were still interested in assessing the putative effect of ceftriaxone on HFS-LTP, as previous reports demonstrate detrimental effects on mossy-fiber-CA3 synaptic plasticity and hippocampal-dependent learning and memory (Omrani et al., 2009; Matos-Ocasio et al., 2014). Here, we focused on HFS-LTP as no impairment in TBS-LTP is evident in Q175FDN heterozygous mice at this age (Quirion and Parsons, 2019). Acute slices from saline or ceftriaxone-treated WT and Q175FDN mice were placed on a multielectrode array (MED64; Fig. 3*A*) and fEPSPs were evoked by electrical stimulation of the Schaffer collaterals. Interestingly, input–output curves revealed larger fEPSP slopes in both genotypes following ceftriaxone treatment (Fig. 3*B–D*; WT-saline, *n* = 5; WT-ceftriaxone, *n* = 8; Q175FDN-saline, *n* = 5; Q175FDN-ceftriaxone, *n* = 8), suggesting that ceftriaxone can increase basal excitability in CA3–CA1. To quantify the putative effects of genotype and ceftriaxone treatment on fEPSP slopes, we graphed the fEPSP slope at 50 μA stimulation (representing an approximately half-maximal stimulus intensity) and analyzed the data using a two-way ANOVA. fEPSPs were not different between WT and Q175FDN mice, although fEPSP slopes were significantly larger following ceftriaxone treatment, independent of genotype (Fig. 3*C*; genotype, *p* = 0.508; treatment, *p* = 0.011; interaction, *p* = 0.906; Sidak’s multiple-comparisons test: *p* = 0.158 for WT-saline vs WT-Cef; *p* = 0.088 for Q175FDN-saline vs Q175FDN-Cef). A significant effect of ceftriaxone was also observed when genotypes were combined for each treatment group (as no genotype effects were noted), and fEPSP slope was plotted against stimulation intensity and analyzed as a two-way ANOVA (Fig. 3*D*; treatment, *p* = 0.005; stimulus intensity, *p* < 0.001; interaction, *p* < 0.001; saline, *n* = 10; ceftriaxone, *n* = 16). Thus, ceftriaxone increases basal excitability at CA3–CA1 synapses. This effect was unlikely to be due to an increase in release probability, as paired-pulse ratios (WT-saline, *n* = 12; Q175FDN-saline, *n* = 6; WT-ceftriaxone, *n* = 10; Q175FDN-ceftraixone, *n* = 5) were not significantly different between WT and Q175FDN mice injected with saline (Fig. 3*E*; repeated-measures two-way ANOVA; genotype, *p* = 0.646; interpulse interval, *p* < 0.001; interaction, *p* = 0.208), between WT and Q175FDN mice injected with ceftriaxone (Fig. 3*F*; repeated-measures two-way ANOVA; genotype, *p* = 0.292; interpulse interval, *p* < 0.001; interaction, *p* = 0.179), or between WT mice injected with saline and WT mice injected with ceftriaxone (Fig. 3*G*; repeated-measures two-way ANOVA; treatment, *p* = 0.497; interpulse interval, *p* < 0.001; interaction, *p* = 0.686). The lack of difference in paired-pulse ratios between WT and Q175FDN mice at this age supports an earlier finding from our laboratory (Quirion and Parsons, 2019).

Next, we evoked LTP using HFS. Q175FDN mice exhibited a lower magnitude of LTP and a significant genotype effect was observed; however, rather than restoring LTP in Q175FDN mice, ceftriaxone had an overall negative impact on LTP (Fig. 3*H–K*; two-way ANOVA; genotype, *p* = 0.015; treatment, *p* = 0.026; interaction, *p* = 0.350; Sidak’s multiple-comparisons test: *p* = 0.054 for WT-saline vs WT-ceftriaxone; *p* = 0.533 for Q175FDN-saline vs Q175FDN-ceftriaxone; WT-saline, *n* = 5; WT-ceftriaxone, *n* = 7; Q175FDN-saline, *n* = 5; Q175FDN-ceftriaxone, *n* = 7). In addition, we also noted that the magnitude of potentiation immediately following HFS appeared to be reduced following ceftriaxone treatment. Thus, we also quantified post-tetanic potentiation (PTP), quantified as the percentage of potentiation within the first 3 min following HFS. PTP represents a form of short-term plasticity that is thought to be mediated by presynaptic protein kinase C and enhanced calcium sensitivity of vesicular release (Brager et al., 2003; Korogod et al., 2007). Indeed, we found that PTP was significantly impaired following ceftriaxone treatment (Fig. 3*H*; two-way ANOVA; genotype, *p* = 0.269; treatment, *p* = 0.010; interaction, *p* = 0.328; Sidak’s multiple-comparisons test: *p* = 0.026 for WT-saline vs WT-ceftriaxone; *p* = 0.358 for Q175FDN-saline vs Q175FDN-ceftriaxone). Together, these data suggest that ceftriaxone treatment can negatively impact hippocampal synaptic plasticity and is therefore unlikely to have a beneficial effect on the cognitive symptoms associated with HD.

### Effect of ceftriaxone on GLT-1 expression is age dependent

**Figure 4.**
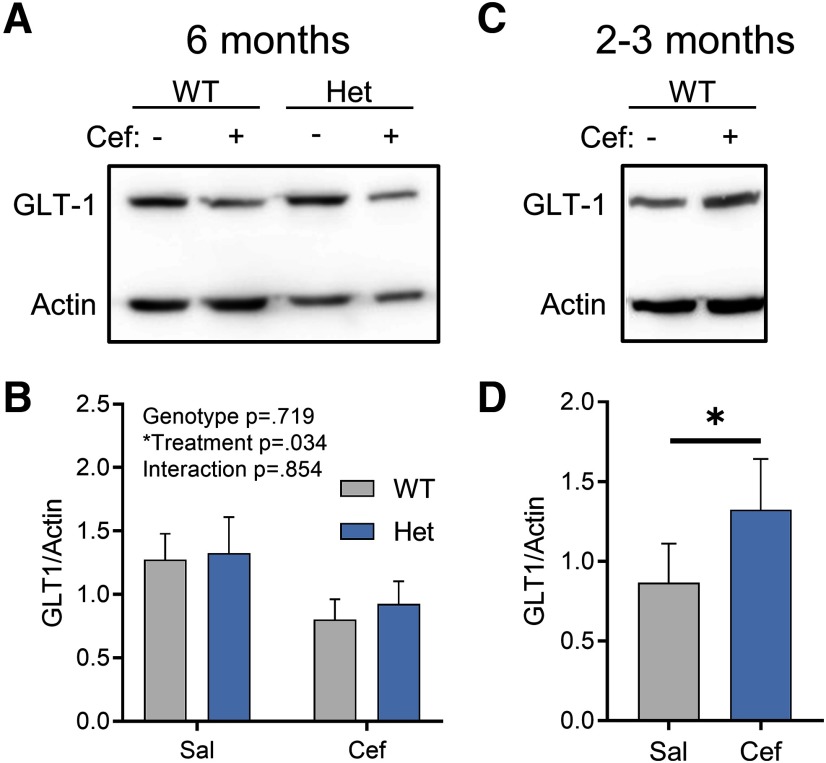
Ceftriaxone exerts an age-dependent effect on hippocampal GLT-1 expression. ***A***, ***B***, Western blots comparing GLT-1 expression in WT and Het mice treated with saline (Sal) or Cef at 6 months of age. ***C***, ***D***, Western blots comparing GLT-1 expression in WT mice treated with Sal or Cef at 2–3 months of age. All data are presented as the mean ± SEM. *p < 0.05.

For each animal used in the aforementioned experiments, hippocampal tissue that was not used for iGluSnFR imaging or electrophysiology was used to quantify GLT-1 expression levels via Western blotting. Surprisingly, we found that in 6-month-old control FVB mice and heterozygous Q175FDN mice, ceftriaxone did not increase GLT-1 expression levels, as expected. In fact, we unexpectedly observed an overall significant effect of ceftriaxone that was due to reduced GLT-1 expression in ceftriaxone-treated mice (Fig. 4*A*; repeated-measured two-way ANOVA; genotype, *p* = 0.719; treatment, *p* = 0.034; interaction, *p* = 0.854; WT-saline, *n* = 7; WT-ceftriaxone, *n* = 7; Q175FDN-saline, *n* = 7; Q175FDN-ceftriaxone, *n* = 7). This overall significant treatment effect was not accompanied by significant *post hoc* tests within either genotype (Sidak’s test: WT-saline vs WT-ceftriaxone, *p* = 0.177; Q175FDN-saline vs Q175FDN-ceftriaxone, *p* = 0.267); thus, the overall significant treatment effect reflects the reduction in mean GLT-1 expression in both genotypes. Moreover, GLT-1 expression was not altered in the Q175FDN hippocampus compared with WT mice, consistent with a previous report in the YAC128 mouse model of HD (Huang et al., 2010). As the majority of studies using ceftriaxone use younger rats or C57BL6/J mice, we reasoned that the inability for ceftriaxone to increase GLT-1 expression in our hands could be due to either mouse strain or age, as the animals used in the present study were 6 month of age. To test the latter, we intraperitoneally injected a separate cohort of 2- to 3-month-old FVB mice with the same ceftriaxone solution and schedule (7 consecutive days) used for the 6-month-old animals. In agreement with previous studies, we now observed a significant increase in GLT-1 expression in ceftriaxone-treated animals compared with saline (paired *t* test, *p* = 0.048; saline, *n* = 5; ceftriaxone, *n* = 5). These results suggest that ceftriaxone has age-dependent effects in FVB/N mice, where increased GLT-1 expression was only observed at younger ages. In all, these data argue against the use of ceftriaxone as a strategy to reduce glutamate toxicity in neurodegenerative disease.

## Discussion

In the present study, using a heterozygous knock-in mouse model of HD at an early symptomatic age, we demonstrated significant alterations in hippocampal extracellular glutamate dynamics that were not alleviated by ceftriaxone treatment. The increased glutamate accumulation observed in Q175FDN hippocampal slices was evident for HFS but not for TBS, suggesting that activity patterns known to induce activity-dependent slowing of glutamate clearance (Armbruster et al., 2016; Pinky et al., 2018) are required for such an effect to be observed. Interestingly, the excessive glutamate levels observed in Q175FDN mice following HFS cannot be explained by low GLT-1 expression levels, as no genotype differences in GLT-1 protein were observed. In our hands, 7 d of 200 mg/kg ceftriaxone increased GLT-1 expression only in younger FVB mice (2–3 months of age) and was unable to increase GLT-1 protein levels in FVB mice 6–7 months of age. Not only did ceftriaxone fail to accelerate extracellular glutamate dynamics, it negatively impacted synaptic plasticity. To the best of our knowledge, this is the first report of altered glutamate dynamics in the HD hippocampus. While such alterations may in part underlie cognitive deficits in HD, our data caution the use of ceftriaxone as a therapeutic option in neurodegenerative disease.

### Altered extracellular glutamate dynamics in the HD hippocampus in response to HFS

Reduced GLT-1 levels have been widely reported in the striatum of HD mouse models and in postmortem striatal tissue from HD patients (Liévens et al., 2001; Behrens et al., 2002; Miller et al., 2008; Faideau et al., 2010). While real-time measurements of iGluSnFR at the population level using wide-field imaging revealed no impairment in glutamate uptake rates in the striatum from YAC128 and R6/2 mouse models of HD (Parsons et al., 2016), a recent study using single-synapse imaging of the ultrafast glutamate sensor iGlu*_u_* variant elegantly demonstrated that a subset of corticostriatal synapses exhibit poor clearance rates in Q175 knock-in mice (Dvorzhak et al., 2019). Here, using the wide-field imaging approach with iGluSnFR, we were able to detect significant alterations in hippocampal extracellular glutamate dynamics in the hippocampus of Q175FDN heterozygous knock-in HD mice at an early symptomatic disease stage (6–7 months; Southwell et al., 2016). Specifically, during HFS, extracellular glutamate accumulation was enhanced in HD tissue as a result of reduced presynaptic depression and a slower clearance rate at HFS offset.

Presynaptic depression that occurs during sustained high-frequency synaptic stimulation (Helassa et al., 2018) contributes to the shape of the overall iGluSnFR response to HFS, and we observed less presynaptic depression in Q175FDN hippocampal tissue compared with WT. Indeed, the degree of ongoing transporter-mediated uptake will also contribute to the shape of the iGluSnFR response during sustained activity; therefore, we also measured iGluSnFR responses to HFS in the presence of the glutamate transporter blocker TBOA. These experiments confirmed that presynaptic release is enhanced during sustained activity in the Q175FDN hippocampus, as HFS-evoked iGluSnFR signals were significantly larger in Q175FDN slices compared with WT slices when glutamate transport was blocked. Huntingtin interacts with numerous proteins involved in regulating presynaptic transmitter release (Shirasaki et al., 2012). While expression levels of presynaptic proteins complexin II, synaptobrevin 2, and rab3A are reduced in the HD striatum, they are enhanced in the HD hippocampus (Morton et al., 2001; Morton and Edwardson, 2001). The elevated expression of presynaptic release machinery in the HD hippocampus may help explain the abnormally high sustain of the iGluSnFR signal to HFS observed in the present study. Our result here is consistent with a recent study that used FM1-43 to quantify synaptic vesicle release in cortical cultures from zQ175 knock-in HD mice. The authors found evidence for enhanced presynaptic calcium influx and vesicle release during sustained field stimulation in zQ175 cultures compared with WT cultures (Chen et al., 2018). Interestingly, age-dependent bidirectional effects on corticostriatal presynaptic release probability have been documented in the YAC128 mouse model of HD, where enhanced release probability at an early age transitions to reduced release probability in later disease stages (Joshi et al., 2009). The present study was conducted at an early symptomatic age in heterozygous Q175FDN mice, so it is possible that the increased iGluSnFR sustain observed may disappear or even reverse at a late disease stage.

We also observed slower glutamate clearance rates following HFS, but not TBS or single pulses, in Q175FDN hippocampal slices. This effect cannot be explained by reduced GLT-1 expression, as total GLT-1 protein was not different between the two genotypes. It is still possible that GLT-1 function is impaired in the Q175FDN hippocampus, as post-translational modifications are known to impact GLT-1 function. In fact, in the YAC128 mouse model of HD, reduced GLT-1 palmitoylation, not expression, was shown to negatively impact glutamate uptake capacity in synaptosomes (Huang et al., 2010). Alternatively, other glutamate transporters such as GLAST and EAAC1 exert significant control over the spatiotemporal dynamics of extracellular glutamate in the hippocampus. For example, a considerable portion of synaptically activated transporter currents, measured by electrophysiological recordings from single astrocytes, remains following complete GLT-1 blockade with a saturating concentration of dihydrokainate (DHK) (Bergles and Jahr, 1997). Similarly, iGluSnFR decay values are magnitudes slower following nonselective transporter blockade with TBOA compared with selective GLT-1 blockade with DHK (Armbruster et al., 2016; Parsons et al., 2016; Pinky et al., 2018). Thus, numerous glutamate transporters combine to shape the overall dynamics of extracellular glutamate in the hippocampus. GLT-1, GLAST (glutamate/aspartate transporter), and EAAC1 exhibit different subcellular localization patterns, with GLT-1 localized to both astrocytes and presynaptic terminals, GLAST localized to astrocytes, and EAAC1 localized to neurons (Rothstein et al., 1994; Lehre et al., 1995; Holmseth et al., 2012). In the present study, slower clearance rates, as measured by iGluSnFR decay, were only observed in the Q175FDN hippocampus in response to HFS, and not to TBS or single pulses. HFS is expected to result in more glutamate spillover compared with TBS or single pulses; thus, it is possible that the clearance of HFS-evoked glutamate release will depend on a different population of transporters than those located in the immediate vicinity of the presynaptic release site. Last, as iGluSnFR decay tau values are determined by sensor kinetics, transporter-mediated uptake as well as diffusion away from the release sites, it is also possible that the volume and architecture of the extracellular space differs between WT and Q175FDN mice. Any putative differences in the tortuosity of the extracellular space (Hrabětová, 2005) or astrocyte-to-neuron proximity (Octeau et al., 2018) may also underlie the observed slower iGluSnFR decay following HFS in the Q175FDN hippocampus.

Recent studies using iGluSnFR have shown a clear dependence of glutamate clearance rate on presynaptic activity (Armbruster et al., 2016; Pinky et al., 2018). This relationship was explored in detail by Armbruster et al. (2016), where they proposed a unique form of neuron–astrocyte communication whereby presynaptic activity exceeding 30 Hz can rapidly and reversibly influence glutamate uptake rates in the cortex. Interestingly, this activity-dependent modulation of glutamate uptake was independent of the amount of glutamate released. It was also demonstrated that while the hippocampus clears glutamate faster than the cortex, hippocampal glutamate dynamics are also slowed by increasing durations of high-frequency presynaptic activity at CA3–CA1 synapses (Pinky et al., 2018). In the cortex, activity-dependent slowing of glutamate clearance is sufficient to slow the decay times of NMDAR-mediated currents (Armbruster et al., 2016). Whether altered glutamate dynamics in the Q175FDN hippocampus translates to slower NMDAR currents remains to be seen and is of interest for future studies. Interestingly, slow clearance rates were only observed after HFS and not after any of the individual bursts associated with TBS. Therefore, instead of a basal deficit in glutamate uptake abilities, it is possible that the activity-dependent slowing of glutamate clearance (Armbruster et al., 2016) may be exaggerated in the HD hippocampus.

### Effects of ceftriaxone on synaptic plasticity, learning, and memory

While ceftriaxone was found to have a beneficial effect on the motor phenotype in the R6/2 mouse model of HD (Miller et al., 2008), its potential effect on learning and memory and/or synaptic plasticity was not investigated in that same study. We were interested in determining the effect of ceftriaxone on CA3–CA1 LTP in the context of HD for the following numerous reasons: (1) the cognitive symptoms of HD are extremely burdensome; (2) hippocampal LTP is impaired in animal models of HD; and (3) ceftriaxone has been shown to negatively impact synaptic plasticity as well as learning and memory, even when administered to healthy animals (Omrani et al., 2009; Matos-Ocasio et al., 2014). We found that ceftriaxone increased basal excitability at CA3–CA1 synapses and negatively impacted synaptic plasticity. The ceftriaxone-induced increase in fEPSP slope could be due to an increase in presynaptic release probability, an increase in synapse numbers, and/or an increase in postsynaptic glutamate receptors. Ceftriaxone was without effect on the paired-pulse ratios in the present study, suggesting that the increase in fEPSP slope may not be due to an increase in release probability. Interestingly, ceftriaxone can increase spine density in the hippocampus (Yang et al., 2013), an effect that may underlie the increase in the postsynaptic population response to electrical stimulation observed in the present study. Ceftriaxone was also shown to impair mossy fiber–CA3 long-term depression (Omrani et al., 2009). Interestingly, in this same study, the authors demonstrated that ceftriaxone had no significant effect on CA3–CA1 LTP, which contrasts with the results of the present study. One possible explanation for this discrepancy is the fact that Omrani et al. (2009) used a stronger LTP induction protocol than the one used here. In such a case, it is possible that ceftriaxone increases the threshold for LTP induction, and, if surpassed, normal LTP can be expressed. The discrepancy may also be explained by age and species differences between the two studies, as Omrani et al. (2009) used male Wistar rats (8–9 weeks old), whereas we used 6-month-old FVB and Q175FDN mice. Nonetheless, the two studies clearly demonstrate that ceftriaxone can have a negative impact on hippocampal synaptic plasticity. On the other hand, it must be acknowledged that multiple studies have indeed demonstrated a beneficial effect of ceftriaxone within the hippocampus of certain disease models (Yimer et al., 2019). For example, daily ceftriaxone injections for 2 months improved memory in the 3xTg mouse model of Alzheimer’s disease (Zumkehr et al., 2015). Thus, the effects of ceftriaxone are complex and likely determined by numerous factors, including treatment duration, brain region, and the particular disease in question. In the case of HD, while ceftriaxone may provide benefit to the striatum, it may have unwanted consequences on hippocampal function.

### Putative age-dependent and GLT-1-independent effects of ceftriaxone

An unexpected finding of the present study was that 7 d of daily ceftriaxone administration (200 mg/kg, i.p.), widely used in the literature, was unable to increase GLT-1 expression in the hippocampus of FVB and Q175FDN mice at 6 months of age. In contrast, the same treatment increased GLT-1 expression in younger FVB mice (2–3 months). This result suggests that it may be more difficult to trigger a ceftriaxone-induced increase in GLT-1 in older animals; indeed, we were unable to find many examples in the literature where a 7 d treatment of ceftriaxone was sufficient to increase GLT-1 expression in aged animals. Five days of ceftriaxone was sufficient to increase GLT-1 expression in WT and R6/2 mice at 13 weeks of age, although later ages were not tested due to the reduced lifespan of the R6/2 mouse model of HD (Sari et al., 2010). While the aforementioned study by Zumkehr et al. (2015) used 10-month-old 3xTg mice, ceftriaxone was administered daily for 2 months. Thus, it is entirely possible that ceftriaxone would exert different effects if the present study was repeated with a longer ceftriaxone dosing period. The effects of ceftriaxone duration and how it affects animals at different ages is of interest for future studies.

Ceftriaxone increases GLT-1 expression by promoting nuclear translocation of p65 and subsequent activation of the transcription factor nuclear factor-κB (Lee et al., 2008). In a previous study, ceftriaxone helped protect acute hippocampal slices from oxygen-glucose deprivation without affecting GLT-1 expression (Lipski et al., 2007). Ceftriaxone has been shown to decrease oxidative stress and neuroinflammation (Yimer et al., 2019) and can also increase expression of the catalytic subunit of the glutamate/cystine antiporter system, which can increase basal glutamate levels (Lewerenz et al., 2009; Trantham-Davidson et al., 2012). The present study provides additional examples of how ceftriaxone can exert significant effects in the brain independent of its widespread reported effect of increasing GLT-1. Thus, the mechanism underlying a reported effect of ceftriaxone, whether it be beneficial or detrimental, should be interpreted with caution and not necessarily attributed exclusively to an increase in GLT-1 expression and an acceleration of transporter-mediated glutamate uptake.

## Synthesis

Reviewing Editor: Quentin Pittman, University of Calgary Decisions are customarily a result of the Reviewing Editor and the peer reviewers coming together and discussing their recommendations until a consensus is reached. When revisions are invited, a fact-based synthesis statement explaining their decision and outlining what is needed to prepare a revision will be listed below. The following reviewer(s) agreed to reveal their identity: Annalisa Scimemi, Chris Dulla.

Both reviewers agree that your study is important and that you are presenting some novel information regarding glutamate handling in the hippocampus. However, both reviewers have raised a number of issues for which you must present additional evidence to assure robustness of the conclusions.

Major:

1. There is a lack of concordance of data illustrations with conclusions and the possible need for additional experiments. For many experiments the n is 5-6, possibly inadequate for arriving at robust conclusions. You should report the statistical power of your tests and if it is low additional experiments should be carried out. The following examples are typical of this problem:

a) Figure 1C - You indicate that ceftriaxone has no effect on iGluSnFR sustain, regardless of genotype. However, in Figure 1C, the magnitude of iGluSnFR sustain in wild type mice after ceftriaxone is half of what it was in baseline conditions (the error bar in ceftriaxone is also roughly half of its value in baseline conditions). Is it possible that there is something wrong with the statistics here?

b) There is a similar concern regarding the experiments described in Figure 2, specifically in Figure 2C. The peak DF/F is clearly different between WT and Q175FDN mice, yet it does not reach statistical significance. There is an obvious discrepancy between data and the conclusions reached by the authors

c) The low n for the Westerns is also concerning; please clarify whether n refers to numbers of animals or number of lanes or slices. Reporting statistical power may help to alleviate concerns as to whether there was an adequate number of samples;

2. If there are differences in HFS- and TBS-induced changes in extracellular glutamate between WT and Q175FDN mice, why did the authors not test the effect of ceftriaxone of TBS-induced LTP? This experiment would help clarify the conclusions about TBS raised by the authors. It would also allow to understand whether different forms of plasticity are equally affected by HD and/or ceftriaxone.

3. The authors postulate that in HD, iGluSnFR increases throughout the duration of an HFS due to increased presynaptic function. An alternative interpretation that the authors don’t consider is that glutamate uptake is decreased in the HD mice allowing glutamate levels to accumulate more. A few simple experiments could clarify this issue. First, the authors could make whole cell recordings from CA1 pyramidal neurons during the HFS and should see increased glutamate release (EPSPs) throughout the duration of the HFS in the HD mice. Alternatively, the authors could block glutamate transporters with TBOA and repeat the HFS protocol. If increased presynaptic function truly drives this finding, then deceased “sustain” seen in the WT animals should be insensitive to TBOA blockade of EAATs while TBOA should have a large effect in the HD mice.

4. What are the iGluSnFR decay times for single stimulations in the HD mice? The authors report decay times for HFS and TBS but not for simple single stimulations. What are the decay times for single stims in HD vs WT hippocampus?

5. Is PPR ratio altered in the hippocampus of HD mice vs WT? Is this data present in the HFS/TBS data the authors already have? Does it suggest a change in Pr?

6. The effects of ceftriaxone of EPSP slope is unexpected. Do the authors think ceftriaxone alters presynaptic function, axonal excitability, or post-synaptic receptor expression. If so, how?

Individual reviewers comments from which the above summary was extracted are appended below:

Reviewer 1

There is evidence that mouse models of Huntington’s disease (HD) express lower levels of the glial glutamate transporter GLT1 in the striatum, and that ceftriaxone (a drug that promotes GLT1 expression) ameliorates the motor deficits in these mice. Since GLT1 is ubiquitously expressed throughout the brain (i.e. not just in the striatum), it is interesting to understand how ceftriaxone affects glutamate homeostasis in other regions of the brain, including the hippocampus. Here, the authors report that ceftriaxone increases hippocampal GLT1 expression only in 2-3 month old FVB mice, not in older mice. They also show that at 6 month of age, ceftriaxone increases cell excitability and decreases HS-LTP equally in WT and Q175FDN mice. Based on these results, they suggest that ceftriaxone may exert different effects in the striatum and hippocampus, and therefore its use as a drug therapy for HD should be cautioned. In general, the manuscript is well written, but there are major concerns about the limited data sets and the statistical power of the comparisons made throughout the text. Expanding the data sets seems to be a priority to support the conclusions made by the authors.

In the methods section, state clearly the strain of mice used for the experiments described in this paper.

Figure 1A - The iGluSnFr fluorescence seems to be mostly confined to hippocampal area CA2. Is this a representative image?

Figure 1C - The authors indicate that ceftriaxone has no effect on iGluSnFR sustain, regardless of genotype. However, in Figure 1C, the magnitude of iGluSnFR sustain in wild type mice after ceftriaxone is half of what it was in baseline conditions (the error bar in ceftriaxone is also roughly half of its value in baseline conditions). Is it possible that there is something wrong with the statistics here?

I have a similar concern regarding the experiments described in Figure 2, specifically in Figure 2C. The peak DF/F is clearly different between WT and Q175FDN mice, yet it does not reach statistical significance. There is an obvious discrepancy between data and the conclusions reached by the authors. One thing that stands out is that for many experiments the n-value is 5-6. I would also like the authors to report the Statistical Power of their tests. If this is low, likely because of the low n-value, I am not sure I can second the data interpretation in its current form.

If there are differences in HFS- and TBS-induced changes in extracellular glutamate between WT and Q175FDN mice, why did the authors not test the effect of ceftriaxone of TBS-induced LTP? This experiment would help clarify the conclusions about TBS raised by the authors. It would also allow to understand whether different forms of plasticity are equally affected by HD and/or ceftriaxone.

I am concerned about the low n-values also for the Western blot experiments. I would like the authors to clarify what exactly n means here. Is it the number of lanes or the number of mice (or maybe both)? Again, reporting the statistical power of this comparison is necessary to understand whether these experiments were performed on a reasonable number of samples.

Reviewer 2:

In this study the authors investigate glutamate signaling in the hippocampus of mice harboring a mutation relevant to Huntington’s disease. Other studies have reported conflicting changes in astrocyte glutamate uptake in a model of HD in the striatum, but little is known about how glutamate uptake is altered in the hippocampus. The authors report a number of interesting findings including altered glutamate accumulation of glutamate in the HD hippocampus during high frequency stimulation, slowed glutamate uptake following HFS in the hippocampus, and several somewhat unexpected findings regarding treating HD animals with ceftriaxone. Overall the paper makes a few new and interesting observations about glutamate handling and the hippocampus of HD, although mechanistic insight is lacking. That said, this is a strong study with meaningful findings. A few key experiments and some clarifications would greatly help strengthen points the authors attempt to make and would increase the impact of the work.

Major comments:

The authors postulate that in HD, iGluSnFR increases throughout the duration of an HFS due to increased presynsaptic function. An alternative interpretation that the authors don’t consider is that glutamate uptake is decreased in the HD mice allowing glutamate levels to accumulate more. A few simple experiments could clarify this issue. First, the authors could make whole cell recordings from CA1 pyramidal neurons during the HFS and should see increased glutamate release throughout the duration of the HFS in the HD mice. Alternatively, the authors could block glutamate transporters with TBOA and repeat the HFS protocol. If increased presynaptic function truly drives this finding, then deceased “sustain” seen in the WT animals should be insensitive to TBOA blockade of EAATs while TBOA should have a large effect in the HD mice.

What are the iGluSnFR decay times altered for single stimulations in the HD mice? The author report decay times for HFS and TBS but not for simple single stimulations. What are the decay times for single stims in HD vs WT hippocampus?

The effects of ceftriaxone of EPSP slope is unexpected. Do the authors think ceftriaxone alters presynaptic function, axonal excitability, or post-synaptic receptor expression. If so, how?

Is PPR ratio altered in the hippocampus of HD mice vs WT? Is this data present in the HFS/TBS data the authors aleady have? Does it suggest a change in Pr?
